# Delayed ethylene glycol poisoning presenting with abdominal pain and multiple cranial and peripheral neuropathies: a case report

**DOI:** 10.1186/1752-1947-4-220

**Published:** 2010-07-21

**Authors:** Fiona Baldwin, Hersharan Sran

**Affiliations:** 1 Department of Intensive Care Medicine, Royal Sussex County Hospital, Brighton, UK

## Abstract

**Introduction:**

Ethylene glycol poisoning may pose diagnostic difficulties if the history of ingestion is not volunteered, or if the presentation is delayed. This is because the biochemical features of high anion-gap metabolic acidosis and an osmolar gap resolve within 24 to 72 hours as the ethylene glycol is metabolized to toxic metabolites. This case illustrates the less well-known clinical features of delayed ethylene glycol poisoning, including multiple cranial and peripheral neuropathies, and the clinical findings which may point towards this diagnosis in the absence of a history of ingestion.

**Case presentation:**

A 53-year-old Afro-Caribbean man presented with vomiting, abdominal pain and oliguria, and was found to have acute renal failure requiring emergency hemofiltration, and raised inflammatory markers. Computed tomography imaging of the abdomen revealed the appearance of bilateral pyelonephritis, however he failed to improve with broad-spectrum antibiotics, and subsequently developed multiple cranial neuropathies and increasing obtundation, necessitating intubation and ventilation. Computed tomography of the brain showed no focal lesions, and a lumbar puncture revealed a raised cerebrospinal fluid opening pressure and cyto-albuminological dissociation. Nerve conduction studies revealed a sensorimotor radiculoneuropathy mimicking a Guillain-Barre type lesion with an atypical distribution. It was only about two weeks after presentation that the history of ethylene glycol ingestion one week before presentation was confirmed. He had a slow recovery on the intensive care unit, requiring renal replacement therapy for eight weeks, and complicated by acute respiratory distress syndrome, neuropathic pain and a slow neurological recovery requiring prolonged rehabilitation.

**Conclusions:**

Although neuropathy as a result of ethylene glycol poisoning has been described in a few case reports, all of these were in the context of a known history of ingestion. As the diagnosis may well be obscured if the history of ingestion is not elucidated, it is important to be aware of this possibility especially if presentation is delayed.

## Introduction

Ethylene glycol (EG) is a common constituent of anti-freeze, coolants and other solvents and is responsible for both inadvertent and intentional poisoning with a reported incidence of exposure in the USA of almost 5000 episodes annually [1]. The diagnosis of EG poisoning is relatively obvious in an individual presenting with a history of ingestion, and a clinical presentation consistent with poisoning, associated with a high anion-gap metabolic acidosis and elevated osmolar gap. EG is metabolized to oxalic acid within 24 to 72 hours and the formation of calcium oxalate monohydrate crystals in the urine, although not specific to the condition, may assist in its elucidation [2].

Even more delayed presentation precludes biochemical evidence of ingestion from the urinalysis, and without a history of exposure, the diagnosis is dependent on anamnesis and a high index of suspicion. There have been previous case reports of neuropathy secondary to EG ingestion, but usually the toxin ingestion is elucidated before the symptoms become apparent, rather than the symptom pattern assisting in the identification of the toxin.

## Case presentation

A 53-year-old Afro-Caribbean man was admitted to another institution with a two-day history of vomiting, hiccups and abdominal pain. He was felt to have gastroenteritis, given intravenous fluids, paracetamol and cyclizine and discharged from the accident and emergency department. Two days later he presented to his general practitioner (GP) with continuing epigastric and lower abdominal pain, vomiting, having not opened his bowels or passed urine for four days.

He had a history of hypercholesterolemia, but was on no regular medications aside from those prescribed two days earlier. He smoked ten cigarettes a day, denied alcohol intake and had no recent travel history. On examination he was apyrexial, clinically dehydrated, with vital signs of pulse rate of 108 per minute and blood pressure of 145/82. Physical examination revealed a generally tender but not peritonitic abdomen and an empty rectum, but was otherwise unremarkable. A urethral catheter was passed and a urine dipstick was strongly positive for blood, protein and less so for glucose and leukocytes.

Laboratory studies revealed a sodium level of 131 mmol/L, potassium level of 6.7 mmol/L, urea of 45.2 mmol/L, and creatinine of 1885 micromol/L (21.3 mg/dL). Of note were a raised C-reactive protein of 421, neutrophil count of 25.9 × 10^9^/L and an erythrocyte sedimentation rate of 100. Blood gases revealed a pH of 7.19, a PO_2 _of 14.8 kPa, pCO_2 _of 5.18 kPa, HCO_3 _of 14.5 mmol/L with a base deficit of -12.9 on 28% inspired oxygen, and an anion gap of 28 mmol/l. An urgent renal ultrasound showed normal sized kidneys with no hydronephrosis and an empty bladder.

He was transferred urgently to the intensive care unit for continuous veno-venous hemodiafiltration and started on ciprofloxacin empirically in view of the history of abdominal pain and raised inflammatory markers.

**Figure 1:**
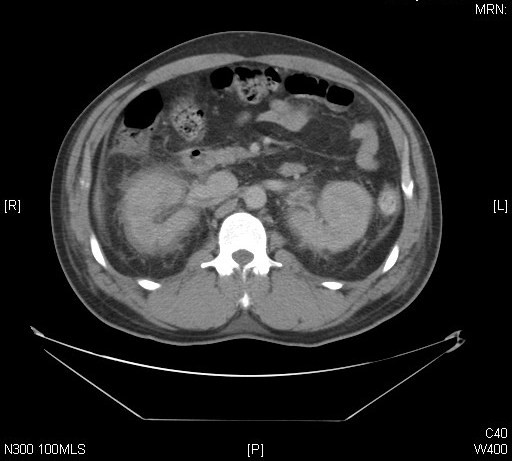
**Computed tomography of abdomen on presentation**.

He was transferred the next day to the renal unit at our centre. A computed tomography (CT) scan of his abdomen showed bilateral perinephric stranding suggestive of pyelonephritis but was otherwise unremarkable (Figure 1). Investigations for underlying causes of the acute renal failure including auto-antibodies, complements and protein electrophoresis were normal.

The patient remained dependent on intermittent hemodialysis. He subsequently complained of back and loin pain, and blurred vision, and was intermittently confused and agitated. On day four of admission he reported having drunk 'battery acid' by mistake one week before his initial presentation, and his family reported a history of cocaine use. At this time there was no focal neurology elicited on examination, and no evidence of mucositis consistent with battery acid ingestion.

**Figure 2:**
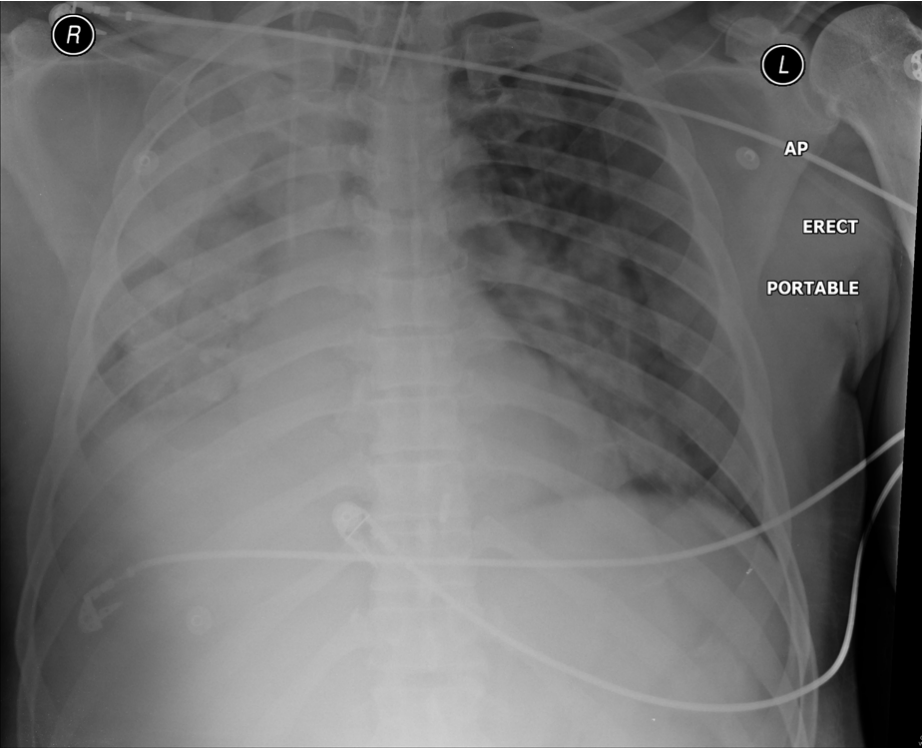
**Chest radiograph on day five**.

On day five of admission he developed respiratory distress due to acute pulmonary edema and possible aspiration pneumonia requiring emergency intubation, ventilation and transfer to the intensive care unit (Figure 2).

Following a good response to antibiotics for aspiration pneumonia, he was extubated within 24 hours, and appeared alert and able to obey commands. Neurological assessment revealed bilateral dilated pupils with sluggish responses to light, and myoclonic jerks of his limbs. Subsequently he became more obtunded and was reintubated for a decreased level of consciousness and hypercapnia.

A CT scan of his brain showed no focal intra-cranial lesion, and a lumbar puncture was performed which showed an elevated opening pressure of 37 cmH_2_0, markedly raised cerebrospinal fluid (CSF) protein at 2785 mg/L but normal CSF white cells 3 × 10^6^/L and no organisms.

He had a tracheostomy inserted and was subsequently co-operative enough to comply with a neurological examination. This revealed bilateral palsies of cranial nerves III, VI and VII, and slow tongue movements but normal palatal movements. He also had distal lower limb weakness with diminished reflexes and down-going plantars and was beginning to complain of distal neuropathic pains. Electromyography and nerve conduction studies revealed a sensorimotor radiculoneuropathy with facial nerve involvement, similar to that seen in Guillain-Barre syndrome, with an atypical distribution.

Faced with a patient with acute renal failure and unexplained neurological disease, a review of the history of toxin ingestion and literature search was performed. The patient remained adamant that he had consumed battery acid, but by this stage he was lucid enough to use his mobile telephone to text his friend to bring in what was by this stage strongly suspected to be anti-freeze. An empty bottle of anti-freeze and coolant was brought in by his friend and the patient confirmed that this was what he had ingested. The product literature confirmed the presence of ethandiol or EG in concentrations of > 90%. As he was anuric for several weeks from admission, it was not possible to analyze a urine specimen for calcium oxalate crystals once this diagnosis became apparent.

Our patient continued to have a rocky course on intensive care complicated by acute respiratory distress syndrome (ARDS), presumed secondary to aspiration pneumonia complicating bulbar palsy, and a slow respiratory wean. He required continuous veno-venous hemofiltration for eight weeks, and is currently independent of dialysis but continues to have a severe degree of renal impairment with an estimated glomerular filtration rate (eGFR) of 13 mL/min. He is likely to require renal replacement therapy in the near future. Neurological recovery was also slow, but he was able to walk again about ten weeks after admission, although rehabilitation was also impaired by marked postural hypotension and persisting neuropathic pain.

## Discussion

This case illustrates the difficulties posed by delayed and concealed EG poisoning. In this case, our patient did not initially volunteer a history of toxin ingestion, and due to the late presentation, initial features such as sedation and inebriation had resolved. The raised anion gap at presentation was presumed secondary to the renal failure and given the delay in presentation and the time course of the excretion of EG, this remains the most likely explanation. Our patient's presenting symptoms of abdominal pain, constipation and anuria, along with the investigations revealing raised inflammatory markers, acute renal failure and an abdominal CT which suggested bilateral pyelonephritis, pointed towards a diagnosis of intra-abdominal sepsis leading to acute renal failure. However, the lack of response to broad-spectrum antibiotics, the history of toxin ingestion and the development of cranial neuropathies pointed towards another diagnosis.

Differential diagnoses considered included a polyneuropathy due to uremia, however this was less likely as the neuropathy developed several days after dialysis was commenced. Other differentials included diabetes mellitus, vasculitis, rheumatoid arthritis, systemic lupus erythematosus, amyloidosis, sarcoidosis, multiple myeloma or other light-chain deposition diseases, and HIV infection, which could also cause renal failure and neuropathies. These differentials were effectively ruled out with the negative autoantibody screen, normal serum electrophoresis and bone marrow biopsy, negative HIV test and normal blood glucose level. Guillain-Barre syndrome was a significant differential; however, once the history of toxin ingestion was elucidated, this became less likely.

Symptoms and signs of EG poisoning are traditionally divided into chronological stages [3]. Stage 1, occurring within 12 hours, consists of central nervous system (CNS) depression, and features of intoxication such as slurred speech and confusion. Stage 2 occurs 12 to 24 hours after ingestion and is characterized by cardiovascular features such as tachycardia, hypertension and hyperventilation due to the production of acid metabolites. Stage 3 occurs after 48 hours and is characterized by acute renal failure [4].

There have been reports in the literature regarding EG and diethylene glycol poisoning causing delayed neurological deficits. However, this phenomenon is still not well described. It may be that, with improvements in medical care, more patients with severe overdoses, which previously would have been fatal, are surviving and therefore displaying these delayed sequelae.

These usually present about one to two weeks after the initial ingestion [4] with cranial neuropathies, including bilateral facial palsy and ophthalmoplegia, as well as peripheral sensorimotor neuropathies which can be severe enough to cause complete paralysis [5]. Lumbar puncture tends to show cytoalbuminological dissociation [6,7], although there can be a CSF leukocytosis [8]. CT brain tends to be normal but magnetic resonance imaging may show abnormal enhancement of cranial nerve nuclei [6,7]. Neurophysiology may show a primary axonal polyneuropathy [5,7], a primary demyelinating pathology [9], or a polyradiculopathy [10,11]. There has been one other case report that mentions the findings of bilateral perinephric stranding on CT in the context of diethylene glycol poisoning [7]. The cases in the literature describe both continued dependence on renal replacement, as well as recovery from acute renal failure, which does not seem to be related to the degree of neurological recovery.

## Conclusions

This case illustrates the diagnostic conundrum that a delayed presentation of EG poisoning may pose. In these situations, therapeutic interventions such as alcohol dehydrogenase inhibition therapy are less of an issue as the offending compound would have been metabolized due to its short half-life. Supportive therapy in terms of renal replacement therapy and ventilatory support for severe neurological deficits are the mainstay of treatment. These patients often have a prolonged course and require extensive rehabilitation.

## Abbreviations

ARDS: acute respiratory distress syndrome; CNS: central nervous system; CSF: cerebrospinal fluid; CT: computed tomography; CVVH: continuous veno-venous hemofiltration; EG: ethylene glycol; eGFR: estimated glomerular filtration rate; GP: general practitioner; ITU: intensive treatment unit; LP: lumbar puncture; MRI: magnetic resonance imaging; SLE: systemic lupus erythematosus.

## Consent

Written informed consent was obtained from the patient for publication of this case report and any accompanying images. A copy of the written consent is available for review by the Editor-in-Chief of this journal.

## Competing interests

The authors declare that they have no competing interests.

## Authors' contributions

Both authors analyzed and interpreted the patient data and the literature review and co-wrote, read and approved the final manuscript.
